# Clearing the air: underestimation of youth smoking prevalence associated with proxy-reporting compared to youth self-report

**DOI:** 10.1186/s12874-022-01594-w

**Published:** 2022-04-11

**Authors:** Eden M. Barrett, Raglan Maddox, Joanne Thandrayen, Emily Banks, Raymond Lovett, Christina Heris, Katherine A. Thurber

**Affiliations:** National Centre for Epidemiology and Population Health, Australian National University, 54 Mills Road, Acton ACT 2601, Australia

**Keywords:** Youth smoking, Survey methods, Proxy reporting, Australia, Tobacco control

## Abstract

**Background:**

Smoking remains a leading cause of disease burden globally. Declining youth smoking prevalence is an essential feature of effective tobacco control; however, accurate data are required to assess progress. This study investigates bias in youth smoking prevalence estimates by respondent type (proxy-reported, self-report with parent present, or self-report independently) for Aboriginal and Torres Strait Islander and total populations of Australia.

**Methods:**

Repeated cross-sectional analysis of representative Aboriginal and Torres Strait Islander Health and National Health Surveys, 2007–2019. Data were restricted to participants aged 15–17 years. Prevalence ratios (PR) and 95% Confidence Intervals (CI) for ever-smoking by respondent type were calculated using Poisson regression with robust standard errors. National youth current-smoking prevalence was estimated if all data were collected by youth self-report; estimates and trends were compared to observed estimates.

**Results:**

Over 75% of all smoking status data were reported by proxy or with parent present. Ever-smoking prevalence among youth self-reporting independently versus proxy-reported was 1.29 (95% CI:0.96–1.73) to 1.99 (95% CI:1.39–2.85) times as high for Aboriginal and Torres Strait Islander youth, and 1.83 (95% CI:0.92–3.63) to 2.72 (95% CI:1.68–4.41) times as high for total population youth. Across surveys, predicted national current-smoking prevalence if all youth self-reported independently was generally higher than observed estimate.

**Conclusions:**

Estimates of youth smoking prevalence are likely inaccurate and underestimated if data are collected by proxy or with parent present. Increased reliance on data reported by youth independently is crucial to improve data accuracy, including to enable accurate assessment of national prevalence.

**Supplementary Information:**

The online version contains supplementary material available at 10.1186/s12874-022-01594-w.

## Background

Tobacco use is a leading contributor to the burden of disease globally, and is an area with substantial potential for health improvement. Effective tobacco control is the combination of reducing tobacco smoking (hereafter referred to as smoking) initiation and increasing cessation in established smokers. Progress in reducing smoking prevalence in Australia is increasingly driven by reduced smoking in youth [1, 2]. Accordingly, youth smoking prevalence is a key outcome measure for national monitoring and evaluation.

Generally, smoking status is accurate when self-reported by the participant [3], particularly if participants perceive a high degree of confidentiality and anonymity in data collection [4]. However, some survey designs allow for youth smoking status to be reported by a proxy (“proxy-reported”), or elicited in the presence of a parent or guardian (“with parent present”).

A proxy reporting on behalf of the youth may not know the actual smoking behaviours. Youth who smoke may be less likely to report smoking with a parent or guardian present, resulting in underreporting. There is limited international evidence on the potential bias in reported youth smoking introduced through use of these data collection methods from international studies [5–7], and none in the Australian context. Progress against Australian policy targets are generally assessed using nationally representative surveys of the Aboriginal and Torres Strait Islander population and of the total population, including national health and social surveys conducted by the Australian Bureau of Statistics (ABS). These surveys collect data on youth smoking through personal interview with the youth, if a parent or guardian consented. Where consent is granted for a personal interview, some youth answer in the presence of a parent or guardian, and others answer with no parent present. Where consent is not granted for a personal interview with the youth, a parent or other adult respond on their behalf (proxy respondent). This may undermine our understanding of true progress in reducing youth smoking, and overall smoking prevalence, in Australia.

We aimed to quantify potential bias by respondent type in youth (15–17 years) smoking prevalence estimates for the Aboriginal and Torres Strait Islander and total population of Australia over time. We aimed to examine the extent to which national youth smoking prevalence estimates and trends could differ from current estimates generated from these surveys if all data were self-reported by youth independently.

## Methods

### Data sources

Existing national cross-sectional surveys conducted by the ABS were accessed through ABS DataLab using Confidential Unit Record Data Files [8]. This included a total of six datasets from surveys conducted between 2007 and 2019; the National Aboriginal and Torres Strait Islander Health Survey (NATSIHS 2018–19), the Australian Aboriginal and Torres Strait Islander Health Survey 2012–13 (AATSIHS 2012–13), and the National Health Survey (NHS, 2007–08, 2011–12, 2014–15, and 2017–18).

These surveys provide representative estimates for the Aboriginal and Torres Strait Islander population (NATSIHS/AATSIHS) and the total population (NHS). Each survey collects information by face-to-face interview from usual residents of private dwellings, covering around 97% of the targeted population. Briefly, the surveys are conducted using a stratified multistage area sample of private dwellings to ensure that all sections of the in-scope population are represented. The NATSIHS comprises a “community” sample, made up of discrete Indigenous communities, and a “non-community” sample, made up of persons in private dwellings in other areas. For the NATSIHS, in each identified Aboriginal and Torres Strait Islander household, up to two adults (≥ 18 years) and two children (0–17 years) were randomly selected in non-remote areas, and up to one adult and one child were randomly selected in remote areas. In the NHS, one adult and one child within each selected dwelling were randomly selected for inclusion. More details on the sampling frame and design of the surveys are available from the ABS [9, 10].

Data were restricted to participants aged 15–17 years as smoking status was not measured for youth younger than 15 years.

### Variables

#### Outcome: smoking status

Youth smoking status was recorded as current daily smoker, current weekly smoker (at least once a week but not daily), current less-than-weekly smoker, ex-smoker, or never-smoker (does not currently smoke, has not previously smoked daily, and has smoked fewer than 100 cigarettes or 20 pipes, cigars or other tobacco products in the participant’s lifetime). Smoking status relates to use of combustible tobacco products only. Participants were categorised as current-smokers (combining daily, weekly, and less-than-weekly), ex-smokers, or never-smokers. A binary ‘ever-smoked’ variable (current- and ex-smoker combined versus never-smoker) and ‘current-smoker’ variable (current-smoker versus ex-smoker and never-smoker combined) was used where required for analysis.

#### Exposure: respondent type

Respondent type was categorised as: proxy-respondent, youth self-report with parent present for some or all smoking questions, or youth self-report independently.

#### Potentially confounding variables

Potentially confounding variables were those factors conceptually considered to be linked to both respondent type and smoking behaviour, restricted to available factors. Sex was categorised as male or female, based on self-reported responses. Age of youth was categorised as 15–16 years or 17 years. Education status of the youth was categorised as currently studying or not currently studying.

Remoteness was categorised as major cities, inner regional, or outer regional and remote for all NHS analyses. Remoteness was categorised as major cities, inner regional, or outer regional, remote and very remote for distribution of respondent type by remoteness in the 2018–19 NATSIHS and the 2012–13 AATSIHS. Tailored distribution data by remoteness were provided by the ABS for the 2012–13 AATSIHS to enable comparability across surveys. Due to use of different remoteness categorisations between datasets, a binary remoteness variable (remote or non-remote) was used as the confounding variable in 2018–19 NATSIHS/2012–13 AATSIHS analyses.

### Statistical analysis

All analyses were repeated for each survey. An alpha level of 0.05 was the threshold for statistical significance. Data were analysed using Stata 16, in ABS DataLab.

#### Unweighted analysis

We quantified the distribution (percentage and 95% Confidence Intervals (CI)) of respondent type for youth smoking data overall and by potentially confounding factors.

The prevalence of current-, ex-, never- and ever-smoking was calculated overall and by respondent type. Prevalence Ratios (PR) and 95% CI for ‘ever-smoked’ by respondent type were calculated in the youth sample of each survey using Poisson regression with robust standard errors. Analyses were adjusted for the potentially confounding factors. Fit of Poisson models was confirmed using Pearson goodness-of-fit test.

#### Weighted analysis

The above analyses were repeated with survey weights applied. For all weighted estimates, data were weighted to the total in-scope population (Aboriginal and Torres Strait Islander or total population), using replicate weights provided by the ABS, and employing the delete-a-group jackknife replication method, described in detail elsewhere [11]. To assess impact of respondent type on estimates of youth smoking status, the PR analysis was also conducted using ‘current-smoker’ as the outcome. These PR results were used to predict the national prevalence of current-smoking if all youth smoking data were collected by youth self-report independently (using the Stata margins command [12], Supplementary table S1). Predicted prevalence estimates and their corresponding 95% CI were compared to those of actual prevalence estimates using an upper tailed Z test. Differences in slope of predicted prevalence and actual prevalence trend lines were compared using methods outlined by Andrade and Perez [13]. Briefly, we tested the assumption of equality of variances between the two regression trend lines using an F-test. The assumption of equality of variances was met; given the small number of time periods, we performed a t-test based on a pooled standard error calculated from the standard errors of the two regression trend lines.

## Results

The substantial majority of youth aged 15–17 years had their smoking status collected by proxy or with parent present (75.6–92.7% of Aboriginal youth and 77.8–86.1% of total population youth) and this proportion generally increased across years examined (Table 1). Youth aged 17 years tended to self-report smoking behaviours independently more often than those aged 15–16 years across both datasets and all years. The distribution across respondent types was generally similar for males and females, by education status and by remoteness across surveys. No participants had missing data for any included variables.

**Table 1:** Distribution of respondent type within the unweighted youth (15–17 years) sample across surveys

	**Respondent type** **Unweighted % (95% CI)**		
	**Proxy**	**Youth self-report with parent present**	**Youth self-report independently**
Aboriginal and Torres Strait Islander population			
2012–13 AATSIHS (*n*** = **757)^1^	37.3 (33.8, 40.7)	38.3 (34.8, 41.8)	24.4 (21.4, 27.5)
Age group			
15–16 years	40.0 (35.8, 44.3)	38.7 (34.4, 42.9)	21.3 (17.7, 24.8)
17 years	31.4 (25.6, 37.3)	37.6 (31.5, 43.6)	31.0 (25.2, 36.8)
Sex			
Male	39.9 (35.0, 44.9)	36.3 (31.5, 41.1)	23.8 (19.5, 28.0)
Female	34.5 (29.7, 39.3)	40.4 (35.4, 45.4)	25.1 (20.7, 29.5)
Remoteness^2^			
Major cities	36.6 (30.1, 43.1)	37.1 (30.6, 43.6)	26.3 (20.4, 32.2)
Inner regional	32.5 (24.3, 40.7)	45.2 (36.5, 53.9)	22.2 (14.9, 29.5)
Outer regional, remote, very remote	39.0 (34.3, 43.7)	36.8 (32.2, 41.4)	24.2 (20.1, 28.3)
Education status			
Currently studying	37.0 (32.7, 41.4)	39.1 (34.7, 43.5)	23.8 (20.0, 27.7)
Not currently studying	37.6 (31.9, 43.3)	36.9 (31.2, 42.6)	25.5 (20.3, 30.6)
2018–19 NATSIHS (*n* = 529)^3^	66.2 (62.1, 70.2)	26.5 (22.7, 30.2)	7.4 (5.1, 9.6)
Age group			
15–16 years	73.2 (68.7, 77.7)	22.3 (18.0, 26.5)	4.6 (2.4, 6.7)
17 years	49.4 (41.5, 57.2)	36.5 (29.0, 44.1)	14.1 (8.6, 19.6)
Sex			
Male	69.3 (63.9, 74.8)	23.1 (18.1, 28.1)	7.6 (4.5, 10.7)
Female	62.7 (56.7, 68.7)	30.2 (24.5, 35.8)	7.1 (4.0, 10.3)
Remoteness			
Major cities	62.7 (54.7, 70.6)	28.9 (21.4, 36.3)	8.5 (3.9, 13.0)
Inner regional	62.9 (53.6, 72.1)	–	–
Outer regional, remote, very remote	69.1 (63.7, 74.6)	–	–
Education status			
Currently studying	67.4 (62.9, 71.9)	25.8 (21.6, 29.9)	6.9 (4.4, 9.3)
Not currently studying	61.3 (52.0, 70.6)	29.2 (20.6, 37.9)	9.4 (3.9, 15.0)
Total population			
2007–08 NHS (*n* = 963)^4^	35.2 (32.2, 38.2)	42.6 (39.4, 45.7)	22.2 (19.6, 24.9)
Age group			
15–16 years	37.2 (33.5, 41.0)	45.1 (41.2, 48.9)	17.7 (14.7, 20.6)
17 years	31.2 (26.1, 36.2)	37.7 (32.4, 42.9)	31.2 (26.1, 36.2)
Sex			
Male	37.3 (32.8, 41.7)	41.0 (36.5, 45.5)	21.7 (17.9, 25.5)
Female	33.3 (29.2, 37.4)	44.0 (39.7, 48.3)	22.7 (19.0, 26.3)
Remoteness			
Major cities	34.9 (31.2, 38.6)	41.8 (38.0, 45.6)	23.3 (20.0, 26.6)
Inner regional	36.4 (30.0, 42.7)	45.5 (38.9, 52.0)	18.2 (13.1, 23.3)
Outer regional, remote	34.6 (25.5, 43.8)	41.3 (31.9, 50.8)	24.0 (15.8, 32.3)
Education status			
Currently studying	35.7 (32.5, 38.9)	43.0 (39.7, 46.3)	21.4 (18.6, 24.1)
Not currently studying	30.9 (21.7, 40.1)	39.2 (29.4, 48.9)	29.9 (20.8, 39.0)
2011–12 NHS (*n* = 937)^5^	30.5 (27.6, 33.5)	48.9 (45.7, 52.1)	20.6 (18.0, 23.2)
Age group			
15–16 years	33.7 (30.0, 37.5)	50.5 (46.5, 54.5)	15.8 (12.9, 18.7)
17 years	24.6 (20.0, 29.3)	45.9 (40.5, 51.3)	29.5 (24.5, 34.4)
Sex			
Male	30.2 (25.9, 34.4)	48.1 (43.4, 52.7)	21.8 (17.9, 25.6)
Female	30.9 (26.8, 34.9)	49.6 (45.2, 54.0)	19.6 (16.1, 23.1)
Remoteness			
Major cities	29.2 (25.6, 32.9)	49.7 (45.7, 53.6)	21.1 (17.8, 24.3)
Inner regional	30.1 (22.9, 37.3)	51.9 (44.1, 59.8)	18.0 (11.9, 24.0)
Outer regional, remote	35.5 (28.3, 42.7)	43.2 (35.7, 50.7)	21.3 (15.1, 27.5)
Education status			
Currently studying	31.1 (28.0, 34.2)	48.9 (45.6, 52.3)	20.0 (17.3, 22.7)
Not currently studying	25.3 (16.3, 34.2)	48.4 (38.1, 58.6)	26.4 (17.3, 35.4)
2014–15 NHS (*n* = 834)^6^	38.0 (34.7, 41.3)	43.4 (40.0, 46.8)	18.6 (15.9, 21.2)
Age group			
15–16 years	39.4 (35.3, 43.4)	46.6 (42.4, 50.7)	14.1 (11.2, 17.0)
17 years	35.4 (29.7, 41.0)	37.1 (31.5, 42.8)	27.5 (22.3, 32.7)
Sex			
Male	40.8 (36.2, 45.5)	44.1 (39.4, 48.8)	15.1 (11.7, 18.5)
Female	35.0 (30.3, 39.7)	42.7 (37.8, 47.5)	22.3 (18.3, 26.4)
Remoteness			
Major cities	37.5 (33.4, 41.7)	44.5 (40.3, 48.8)	17.9 (14.7, 21.2)
Inner regional	32.7 (25.7, 39.8)	45.0 (37.6, 52.5)	22.2 (16.0, 28.5)
Outer regional, remote	46.6 (38.1, 55.1)	36.8 (28.6, 45.1)	16.5 (10.2, 22.9)
Education status			
Currently studying	38.2 (34.8, 41.6)	43.8 (40.3, 47.3)	18.0 (15.3, 20.7)
Not currently studying	34.7 (21.3, 48.0)	36.7 (23.2, 50.3)	28.6 (15.9, 41.2)
2017–18 NHS (*n* = 878)^7^	46.5 (43.2, 49.8)	39.6 (36.4, 42.9)	13.9 (11.6, 16.2)
Age group			
15–16 years	48.5 (44.3, 52.6)	39.9 (35.8, 44.0)	11.6 (9.0, 14.3)
17 years	43.0 (37.5, 48.4)	39.2 (33.8, 44.5)	17.9 (13.7, 22.1)
Sex			
Male	49.1 (44.4, 53.8)	38.4 (33.8, 42.9)	12.6 (9.4, 15.7)
Female	43.9 (39.2, 48.5)	40.9 (36.3, 45.5)	15.2 (11.9, 18.6)
Remoteness			
Major cities	44.5 (40.3, 48.7)	42.4 (38.3, 46.6)	13.1 (10.3, 15.9)
Inner regional	52.9 (45.4, 60.5)	31.8 (24.8, 38.8)	15.3 (9.9, 20.7)
Outer regional, remote	46.4 (38.8, 54.0)	38.6 (31.1, 46.0)	15.1 (9.6, 20.5)
Education status			
Currently studying	46.9 (43.5, 50.4)	39.7 (36.3, 43.0)	13.4 (11.0, 15.7)
Not currently studying	40.6 (28.6, 52.7)	39.1 (27.1, 51.0)	20.3 (10.4, 30.2)

– indicates that data were not presented due to small numbers in one or more categories

CI, confidence intervals; NATSIHS, National Aboriginal and Torres Strait Islander Health Survey; NHS, National Health Survey

1. Australian Bureau of Statistics (2020) Australian Aboriginal and Torres Strait Islander Health Survey (Core component) 2012–13, DataLab

2. Australian Bureau of Statistics, Australian Statistical Geography Standard Remoteness Structure, ABS, Canberra. Available: https://www.abs.gov.au/websitedbs/d3310114.nsf/home/remoteness+structure

3. Australian Bureau of Statistics (2020) National Aboriginal and Torres Strait Islander Health Survey, 2018–19, DataLab

4. Australian Bureau of Statistics (2020) National Health Survey 2007–08, DataLab

5. Australian Bureau of Statistics (2020) National Health Survey 2011–12, DataLab

6. Australian Bureau of Statistics (2020) National Health Survey 2014–15, DataLab

7. Australian Bureau of Statistics (2020) National Health Survey 2017–18, DataLab

Among Aboriginal and Torres Strait Islander youth with proxy-reported smoking status 21.3% (95% CI 16.5–26.1) were current-smokers in 2012/13 and 17.4% (95% CI 13.4–21.4) in 2018/19, compared to 25.4% (95% CI 19.1–31.7) and 33.3% (95% CI 18.5–48.2) of youth self-reporting independently in the corresponding years (Table 2). In the total population youth sample, current-smoking prevalence was 4.7% (95% CI 2.5–7.0) in 2007/08 and 5.6% (95% CI 2.9–8.3) in 2011/12 among youth with proxy-reported smoking status, compared to 15.4% (95% CI 10.6–20.3; 2007/8) and 12.4% (95% CI 7.8–17.1; 2011/12) among youth self-reporting independently.

**Table 2:** Prevalence of current, ex-, never, and ever smokers overall and by respondent type, and aPRs for ‘ever smoked’ by respondent type, within the youth (15–17 years) sample of each survey

	**Smoking status** **Unweighted % (95% CI)**				**Ever smoked**
	**Current smoker**	**Ex-smoker**	**Never smoker**	**Ever smoker**	**aPR (95% CI)**
**Aboriginal and Torres Strait Islander population**					
**2012–13 AATSIHS**^**1**^					
Total	23.0 (20.0, 26.0)	3.7 (2.4, 5.0)	73.3 (70.2, 76.5)	26.7 (23.5, 29.8)	-
Proxy	21.3 (16.5, 26.1)	–	–	–	1 (Ref)
Youth self-report with parent present	23.1 (18.2, 28.0)	4.5 (2.1, 6.9)	72.4 (67.3, 77.6)	27.6 (22.4, 32.7)	1.24 (0.94, 1.63)
Youth self-report independently	25.4 (19.1, 31.7)	–	–	–	1.29 (0.96, 1.73)
**2018–19 NATSIHS**^**2**^					
Total	19.3 (15.9, 22.7)	3.8 (2.2, 5.4)	76.9 (73.3, 80.5)	23.1 (19.5, 26.7)	-
Proxy	17.4 (13.4, 21.4)	–	–	–	1 (Ref)
Youth self-report with parent present	20.0 (13.4, 26.6)	7.9 (3.4, 12.3)	72.1 (64.7, 79.6)	27.9 (20.4, 35.3)	1.45 (1.01, 2.07)
Youth self-report independently	33.3 (18.5, 48.2)	–	–	–	1.99 (1.39, 2.85)
**Total population**					
**2007–08 NHS**^**3**^					
Total	7.4 (5.7, 9.0)	3.9 (2.7, 5.2)	88.7 (74.0, 84.9)	11.3 (9.3, 13.3)	-
Proxy	4.7 (2.5, 7.0)	–	–	–	1 (Ref)
Youth self-report with parent present	5.4 (3.2, 7.5)	–	–	–	1.58 (0.98, 2.56)
Youth self-report independently	15.4 (10.6, 20.3)	5.1 (2.2, 8.1)	79.4 (74.0, 84.9)	20.6 (15.1, 26.0)	2.72 (1.68, 4.41)
**2011–12 NHS**^**4**^					
Total	7.3 (5.6, 8.9)	4.2 (2.9, 5.4)	88.6 (86.5, 90.6)	11.4 (9.4, 13.5)	-
Proxy	5.6 (2.9, 8.3)	–	–	–	1 (Ref)
Youth self-report with parent present	6.1 (3.9, 8.3)	–	–	–	1.65 (1.01, 2.72)
Youth self-report independently	12.4 (7.8, 17.1)	6.7 (3.2, 10.3)	80.8 (75.3, 86.4)	19.2 (13.6, 24.7)	2.71 (1.59, 4.62)
**2014–15 NHS**^**5**^					
Total	3.8 (2.5, 5.1)	2.5 (1.5, 3.6)	93.6 (92.0, 95.3)	6.4 (4.7, 8.0)	-
Proxy	–	–	95.6 (93.3, 97.8)	4.4 (2.5, 6.9)	1 (Ref)
Youth self-report with parent present	–	–	94.5 (92.1, 96.8)	5.5 (3.2, 7.9)	1.22 (0.64, 2.31)
Youth self-report independently	–	–	87.7 (82.6, 92.9)	12.3 (7.1, 17.4)	2.31 (1.19, 4.48)
**2017–18 NHS**^**6**^					
Total	3.2 (2.0, 4.4)	2.4 (1.4, 3.4)	94.4 (92.9, 95.9)	5.6 (4.1, 7.1)	-
Proxy	–	–	95.3 (93.3, 97.4)	4.7 (2.6, 6.7)	1 (Ref)
Youth self-report with parent present	–	–	94.8 (92.5, 97.2)	5.2 (2.8, 7.5)	1.12 (0.60, 2.07)
Youth self-report independently	–	–	90.2 (84.9, 95.5)	9.8 (4.5, 15.1)	1.83 (0.92, 3.63)

– indicates that data were not presented due to small numbers in one or more categories. Prevalence estimates and aPRs are calculated using unweighted data

aPR, adjusted prevalence ratio (adjusted for age, sex, remoteness and education status); CI, confidence intervals; NATSIHS, National Aboriginal and Torres Strait Islander Health Survey; NHS, National Health Survey

1. Australian Bureau of Statistics (2020) Australian Aboriginal and Torres Strait Islander Health Survey (Core component) 2012–13, DataLab

2. Australian Bureau of Statistics (2020) National Aboriginal and Torres Strait Islander Health Survey, 2018–19, DataLab

3. Australian Bureau of Statistics (2020) National Health Survey 2007–08, DataLab

4. Australian Bureau of Statistics (2020) National Health Survey 2011–12, DataLab

5. Australian Bureau of Statistics (2020) National Health Survey 2014–15, DataLab

6. Australian Bureau of Statistics (2020) National Health Survey 2017–18, DataLab

Ever-smoking prevalence was 1.29 (95% CI 0.96–1.73; 2012/13) and 1.99 (95% CI 1.39–2.85; 2018/19) times as high in Aboriginal and Torres Strait Islander youth who self-reported smoking status independently compared to those with proxy-reported smoking status (Table 2). Similarly, in the total population youth sample, the prevalence across all surveys of ever-smoking was 1.83 (95% CI 0.92–3.63; 2017/18) to 2.72 (95% CI 1.68–4.41; 2007/08) times as high in youth who-self reported smoking status independently compared to those with proxy-reported smoking status. The association was significant in all surveys except the 2012/13 AATSIHS and the 2017/18 NHS. Generally, ever-smoking prevalence was similar or slightly higher for youth self-reporting with a parent present compared to youth with proxy-reported smoking status; it was significantly higher for two surveys, the 2018/19 NATSIHS (PR 1.45; 95% CI 1.01–2.07) and the 2011/12 NHS (PR 1.65; 95% CI 1.01–2.72). Patterns were similar using weighted data (Supplementary Table S2-S3).

Current-smoking prevalence among youth self-reporting independently versus report-by-proxy was 1.52 (95% CI 0.92–2.51; 2012/13) to 1.73 (95% CI 0.73–4.05; 2018/19) times as high for Aboriginal and Torres Strait Islander youth, and 1.37 (95% CI 0.49–3.85; 2017/18) to 4.85 (95% CI 1.86–12.62; 2007/08) times as high for total population youth. While consistent with higher current-smoking prevalence among youth reporting independently, CIs were wide due to small numbers of current-smokers, and the association was only significant for the 2007/08 NHS (Supplementary Table S1).

**Fig. 1:**
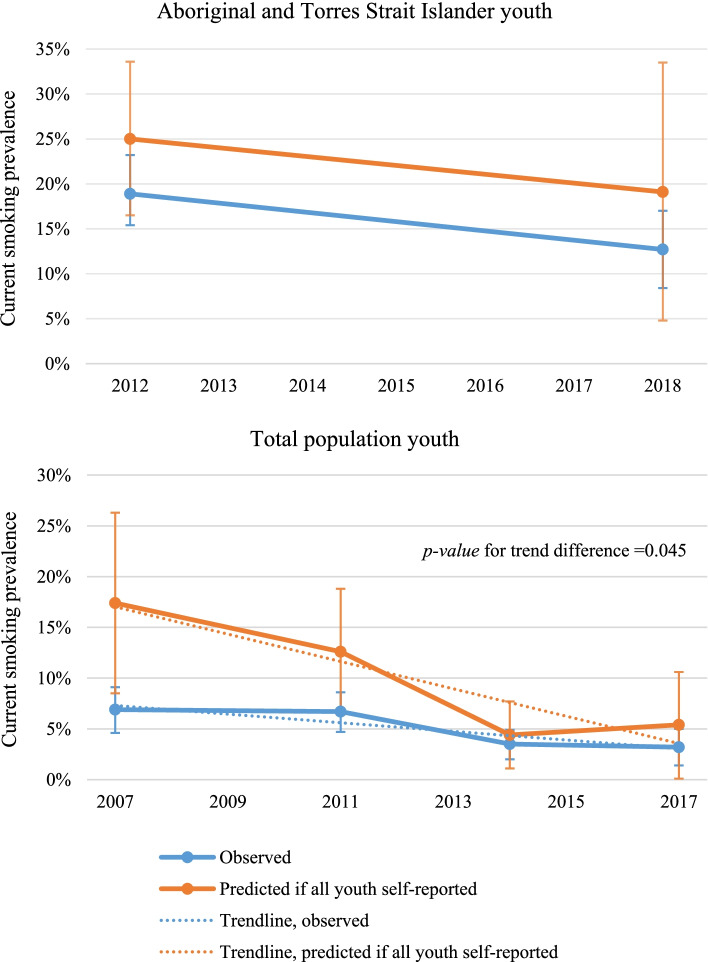
Observed and predicted national current-smoking prevalence if all youth (aged 15–17 years) self-reported independently.

In all six surveys, the predicted national current-smoking prevalence if all youth were to self-report smoking status independently was substantially higher than the observed estimate based on actual responses (Fig. 1; Supplementary table S1). The difference was significant in the 2007/08 (17.4% vs 6.9% respectively, *p-value* = 0.011) and 2011/12 NHS (12.6% vs 6.7%, *p-value* = 0.035).

See Table S3 for the underlying data. 2012: Australian Bureau of Statistics, Australian Aboriginal and Torres Strait Islander Health Survey (Core component) 2012-13, (accessed through Australian Bureau of Statistics DataLab July 2021).  2018: Australian Bureau of Statistics, National Aboriginal and Torres Strait Islander Health Survey, 2018-19, (accessed through Australian Bureau of Statistics DataLab July 2021).  2007: Australian Bureau of Statistics, National Health Survey 2007-08, (accessed through Australian Bureau of Statistics DataLab July 2021).  2011: Australian Bureau of Statistics, National Health Survey 2007-08, (accessed through Australian Bureau of Statistics DataLab July 2021).  2014: Australian Bureau of Statistics, National Health Survey 2014-15, (accessed through Australian Bureau of Statistics DataLab July 2021).  2017: Australian Bureau of Statistics, National Health Survey 2017-18, (accessed through Australian Bureau of Statistics DataLab July 2021).  

The rate of change in Aboriginal and Torres Strait Islander youth current-smoking was similar using observed data and predicted estimates if all youth self-reported independently while the rate of change in total population youth was significantly greater using the predicted estimate if all youth self-reported independently (*p-value* = 0.045) (Fig. 1).

## Discussion

One of Australia’s largest sources of representative data about youth smoking prevalence predominantly, and increasingly over time, relies on data about youth behaviours reported by proxy or with an adult present at interview. Data collection by proxy or with parental presence leads to under-reporting compared to youth self-report independently and is likely to have resulted in underestimation of actual national youth smoking prevalence from 2007–2019, with predicted prevalence 1.3 to 2.5 times as high as the observed prevalence if all youth had self-reported. Based on the most recent survey data (2018–19 NATSIHS and 2017–18 NHS), there may be up to 3200 more Aboriginal and Torres Strait Islander youth and 18,600 more total population youth currently smoking than estimated based on observed responses. There is potential for further increasing bias in national health surveys if the percentage of youth self-reporting independently continues to decline.

In Australia’s total population youth, the gap between predicted and observed smoking prevalence narrowed over time, reflecting changes in the proportion of youth self-reporting independently, bias (current smoking prevalence ratio for independent report vs proxy report) at each time point, and actual smoking behaviour. If all youth had self-reported independently, we may have seen a similar absolute prevalence decline in Aboriginal and Torres Strait Islander youth (5.9% compared to 6.2% in observed data). In total population youth, we may have seen a larger absolute prevalence decline over time (12.0% compared to 3.7% in observed data), but with a much higher starting point.

These findings and the observed magnitude of association are in accordance with the limited international evidence which suggest that youth smoking data reported by a parent proxy [6], or in the presence of a parent [7], results in underreporting of youth smoking. Harakeh et al. found that the percentage of youth aged 14–17 years who had ever tried smoking in a sample from the Netherlands was nearly double when self-reported by the youth independently versus proxy-reported by the mother (47.8% vs 26.8%)[6]. In a representative sample of Californian students aged 12–17 years, parental presence at data collection was associated with 30% lower odds of reporting current-smoking (OR 0.70, 95% CI: 0.56,0.86)[7]. Collection of other forms of data, including alcohol and drug use and e-cigarettes, by parent proxy or with parent present show similar results [14–16].

Collecting precise data on smoking behaviour is critical to monitor smoking trends over time and is consistent with the World Health Organization’s Framework Convention on Tobacco Control [17], signed by Australia, which states that “*each party shall endeavour to: …progressively establish and maintain updated data from national surveillance programmes…*” (pg. 18). This is particularly important given youth non-uptake is vital to the success of tobacco control. The Implementation Plan for the National Aboriginal and Torres Strait Islander Health Plan 2013–2023 [2] sets targets to increase the prevalence of Aboriginal and Torres Strait Islander youth aged 15–17 years who have never smoked from 77 to 91% by 2023. Smoking population prevalence data from the ABS national health and social surveys are used to inform progress against the Implementation Plan target, and could also inform the imminent new Implementation Plan for the National Aboriginal and Torres Strait Islander Health Plan, the next iteration of the National Tobacco Strategy and the National Preventative Health Strategy. Underestimating youth smoking due to bias within these surveys may lead to a false sense of security regarding tobacco control, particularly if reliance on proxy report continues to increase. This may also lead to an artificial jump in smoking prevalence at age 18 years when survey participants all self-report independently.

It is prudent to consider alternative methods for collecting data from youth within national surveys. Strategies such as gaining consent for the youth to self-report privately using computer-assisted self-interviewing software have been employed within other surveys in efforts to ensure greater privacy in youth data collection [18]. Further research is warranted to explore if this method could be used to produce more valid youth smoking data, including their feasibility within large quantitative survey data collection.

Within Australia, other sources of youth smoking data are also used to monitor trends, although each has its own potential limitations. The Australian Secondary Students’ Alcohol and Drug Survey (ASSAD) collects data on youth smoking every three years from a representative sample of Australian students (aged 12–17 years) enrolled in school nationally [19]. The ASSAD is administered on school premises, which has been shown to result in higher reported youth smoking compared to surveys administered in the home [4, 7]. However, the sample is restricted to youth who attend school, and does not adequately capture youth living in remote areas, or attending small schools (fewer than 100 students) [20]. These limits in survey scope are likely to lead to an underestimation of smoking prevalence, particularly in the Aboriginal and Torres Strait Islander youth population. The National Drug Strategy Household Survey (NDSHS) has collected data on youth smoking behaviours every three years since 1985. Although the NDSHS does not collect youth smoking data by proxy, it does allow parents to be present at data collection. In the 2019 NDNHS, parent presence for youth aged 14–15 years was around 40% [21]. Like the ASSAD, the NDSHS should not be used for Aboriginal and Torres Strait Islander-specific estimates. Australia currently lacks any other nationally representative data about Aboriginal and Torres Strait Islander youth (15–17 years) smoking, resulting in a reliance solely on the ABS national health and social surveys. A detailed overview of other sources of youth smoking data in Australia has been included in Supplementary Material (Table S4).

There are several limitations to consider in the interpretation of these findings. The changes across ABS surveys in the scope, sample design, coverage, and questions asked and category definitions make it difficult to confidently assess trends in smoking. The prevalence estimates presented here may contrast with the findings of other analyses that used different categorisations of smoking status or age groups. This problem is compounded by the limited number of Aboriginal and Torres Strait Islander surveys with data on respondent type available. For example, data from another ABS survey, the National Aboriginal and Torres Strait Islander Social Survey (NATSISS), also collects youth smoking data by proxy or with parent present, and is also used to monitor trends in Aboriginal and Torres Strait Islander youth smoking. However, data on respondent type is unavailable for the NATSISS, and could not be included in the analysis.

Additional factors such as the youth’s level of independence and the smoking status of the parent present, not measured in this dataset, may relate to parent presence at interview and youth smoking behaviours. These factors may influence the extent to which youth are comfortable disclosing their actual smoking behaviours. However, any additional factors are unlikely to account for the magnitude of difference observed.

Lastly, we assume youth smoking data when self-reported independently is more likely to be correct. There are additional factors that can introduce bias in self-report, as even specific characteristics of the interviewer can influence responses [22]. The reliability of this self-reported youth smoking data was not validated with biochemical measures. However, previous research has shown that smoking in adolescents can be accurately assessed with self-reports if confidentiality and anonymity are guaranteed [3, 23], as is more likely the case when youth self-report independently.

## Conclusion

Our findings demonstrate that youth tobacco smoking estimates are unlikely to be accurate if drawn from data collected by a proxy respondent or with parent present. In order to improve the accuracy of data on youth smoking behaviours, it is important to collect sufficient data through self-report, in a safe and confidential manner. To achieve this within these Australian ABS surveys would require both increasing the number of youth who are present at interview, and increasing parents’ willingness to have the youth self-report independently. Furthermore, it is critical to assess the suitability of available data sources for measuring and monitoring prevalence and trends in youth smoking.

## Supplementary Information

**Additional file 1.** 

## Data Availability

EMB and KAT had full access to all of the data (including statistical reports and tables) in the study through Australian Bureau of Statistics (ABS) DataLab. All authors had access to all aggregated results cleared for release from ABS DataLab. JT conducted statistical analysis on aggregated results. The unit-record survey data are available to researchers, in accordance with ABS data access procedures and policies. More information is available at the ABS website: https://www.abs.gov.au/websitedbs/d3310114.nsf/home/microdata+entry+page.

## Abbreviations

AATSIHS: Australian Aboriginal and Torres Strait Islander Health Survey
ABS: Australian Bureau of Statistics
ASSAD: Australian Secondary Students’ Alcohol and Drug Survey
CI: Confidence Intervals
NATSIHS: National Aboriginal and Torres Strait Islander Health Survey
NATSISS: National Aboriginal and Torres Strait Islander Social Survey
NDSHS: National Drug Strategy Household Survey
NHS: National Health Survey
PR: Prevalence Ratios
