# Supplementary material for: Clearing the air: underestimation of youth smoking prevalence associated with proxy-reporting compared to youth self-report
Source: BMC Med Res Methodol. 2022 Apr 11;22:108. doi: 10.1186/s12874-022-01594-w (PMC8996602; doi:10.1186/s12874-022-01594-w)
Supplement: Supplementary file 1 — Additional file 1. [file 12874_2022_1594_MOESM1_ESM.docx]

## Supplementary File

**Table S1. Adjusted Prevalence Ratios (aPRs) for youth (15-17 years) ‘current smoker’ by respondent type using weighted data, national youth current-smoking prevalence based on observed data, and predicted national youth current-smoking prevalence if all youth self-reported independently**

|  | **Current smoker** | **National current smoker prevalence estimates** |  |  |
| --- | --- | --- | --- | --- |
|  | **aPR (95% CI)** | **Observed data**  **Weighted % (95% CI)** | **If all youth self-reported independently**  **Weighted % (95% CI)** | ***p-value*** |
| **Aboriginal and Torres Strait Islander population** |  |  |  |  |
| **2012-13 AATSIHS^1^** |  |  |  |  |
| Total | - | 18.9 (15.4, 22.3) | 25.0 (16.5, 33.6) | 0.095 |
| Proxy | 1 (Ref) |  |  |  |
| Youth self-report with parent present | 1.07 (0.67, 1.71) |  |  |  |
| Youth self-report independently | 1.52 (0.92, 2.51) |  |  |  |
| **2018-19 NATSIHS^2^** |  |  |  |  |
| Total | **-** | 12.7 (8.4, 17.0) | 19.1 (4.8, 33.5) | 0.198 |
| Proxy | 1 (Ref) |  |  |  |
| Youth self-report with parent present | 0.73 (0.29,1.88) |  |  |  |
| Youth self-report independently | 1.73 (0.73,4.05) |  |  |  |
| **Total population** |  |  |  |  |
| **2007-08 NHS^3^** |  |  |  |  |
| Total | - | 6.9 (4.6, 9.1) | 17.4 (8.5, 26.3) | 0.011 |
| Proxy | 1 (Ref) |  |  |  |
| Youth self-report with parent present | 1.32 (0.47, 3.74) |  |  |  |
| Youth self-report independently | 4.85 (1.86, 12.62) |  |  |  |
| **2011-12 NHS^4^** |  |  |  |  |
| Total | - | 6.7 (4.7, 8.6) | 12.6 (6.4, 18.8) | 0.035 |
| Proxy | 1 (Ref) |  |  |  |
| Youth self-report with parent present | 0.98 (0.41, 2.35) |  |  |  |
| Youth self-report independently | 2.43 (0.92, 6.39) |  |  |  |
| **2014-15 NHS^5^** |  |  |  |  |
| Total | - | 3.5 (2.0, 4.9) | 4.4 (1.1, 7.7) | 0.294 |
| Proxy | 1 (Ref) |  |  |  |
| Youth self-report with parent present | 1.03 (0.38, 2.82) |  |  |  |
| Youth self-report independently | 1.44 (0.49, 4.22) |  |  |  |
| **2017-18 NHS^6^** |  |  |  |  |
| Total | - | 3.2 (1.4, 5.0) | 5.4 (0.1, 10.6) | 0.219 |
| Proxy | 1 (Ref) |  |  |  |
| Youth self-report with parent present | 0.40 (0.10, 1.59) |  |  |  |
| Youth self-report independently | 1.37 (0.49, 3.85) |  |  |  |

aPR = Prevalence Ratio adjusted for age, sex, remoteness, and education status. Prevalence Ratios and prevalence estimates are calculated using weighted data. The p-values presented (calculated using upper tailed Z-test) are from the statistical test of difference between national current smoker prevalence estimated using observed data compared to if all youth self-reported independently.

CI, confidence intervals; NATSIHS, National Aboriginal and Torres Strait Islander Health Survey; NHS, National Health Survey.

1. Australian Bureau of Statistics, Australian Aboriginal and Torres Strait Islander Health Survey (Core component) 2012-13, (accessed through Australian Bureau of Statistics DataLab July 2021).

2. Australian Bureau of Statistics, National Aboriginal and Torres Strait Islander Health Survey, 2018-19, (accessed through Australian Bureau of Statistics DataLab July 2021).

3. Australian Bureau of Statistics, National Health Survey 2007-08, (accessed through Australian Bureau of Statistics DataLab July 2021).

4. Australian Bureau of Statistics, National Health Survey 2011-12, (accessed through Australian Bureau of Statistics DataLab July 2021).

5. Australian Bureau of Statistics, National Health Survey 2014-15, (accessed through Australian Bureau of Statistics DataLab July 2021).

6. Australian Bureau of Statistics, National Health Survey 2017-18, (accessed through Australian Bureau of Statistics DataLab July 2021).

**Table S2. Distribution of respondent type within the weighted youth population (aged 15-17 years) across surveys, overall and by age, sex, remoteness, and education status**

|  | **Respondent type**  **Weighted % (95% CI)** |  |  |
| --- | --- | --- | --- |
|  | **Proxy** | **Youth self-report with parent present** | **Youth self-report independently** |
| **Aboriginal and Torres Strait Islander population** |  |  |  |
| **2012-13 AATSIHS^1^** | **36.5 (31.9, 41.2)** | **40.4 (35.5, 45.3)** | **23.1 (19.3, 26.8)** |
| **Age group** |  |  |  |
| 15-16 years | 39.5 (33.1, 45.8) | 40.6 (33.8, 47.4) | 19.9 (15.5, 24.3) |
| 17 years | 30.1 (21.2, 39.0) | 40.0 (29.6, 50.5) | 29.9 (22.0, 37.8) |
| **Sex** |  |  |  |
| Male | 37.7 (31.3, 44.0) | 39.7 (32.8, 46.6) | 22.7 (17.5, 27.8) |
| Female | 35.3 (28.5, 42.1) | 41.2 (34.4, 48.1) | 23.5 (18.2, 28.8) |
| **Remoteness^2^** |  |  |  |
| Major cities | 36.6 (29.2, 44.0) | 37.4 (29.4, 45.4) | 26.0 (19.4 32.6) |
| Inner regional | 32.4 (21.9, 42.9) | 47.7 (37.5, 57.9) | 19.9 (13.4, 26.4) |
| Outer regional, remote, very remote | 38.8 (31.2, 46.5) | 38.9 (30.7, 47.1) | 22.2 (15.9, 28.6) |
| **Education status** |  |  |  |
| Currently studying | 37.9 (31.5, 44.4) | 38.8 (31.5, 46.0) | 23.3 (18.3, 28.3) |
| Not currently studying | 33.9 (25.7, 42.1) | 43.5 (31.2, 55.7) | 22.6 (15.7, 29.6) |
| **2018-19 NATSIHS^3^** | **64.9 (56.8, 72.9)** | **28.6 (20.2, 37.1)** | **6.5 (2.8, 10.2)** |
| **Age group** |  |  |  |
| 15-16 years | 73.4 (64.9, 81.9) | 23.8 (15.2, 32.4) | 2.8 (0.2, 5.5) |
| 17 years | 40.9 (23.9, 57.8) | 42.3 (24.4, 60.3) | 16.8 (5.8, 27.8) |
| **Sex** |  |  |  |
| Male | 71.1 (60.8, 81.3) | 23.6 (13.0, 34.2) | 5.3 (1.3, 9.4) |
| Female | 58.3 (47.4, 69.2) | 33.9 (22.7, 45.2) | 7.7 (1.6, 13.9) |
| **Remoteness** |  |  |  |
| Major cities | 77.5 (65.9, 89.1) | 20.8 (9.3, 32.3) | 1.7 (0.2, 3.2) |
| Inner regional | 51.1 (28.5, 73.8) | -- | -- |
| Outer regional, remote, very remote | 61.4 (51.7, 71.1) | -- | -- |
| **Education status** |  |  |  |
| Currently studying | 67.2 (58.6, 75.7) | 27.1 (18.1, 36.0) | 5.8 (2.0, 9.5) |
| Not currently studying | 55.2 (33.6, 76.8) | 35.2 (13.4, 57.0) | 9.6 (0.0, 19.5) |
| **Total population** |  |  |  |
| **2007-08 NHS^4^** | **35.9 (31.2, 40.5)** | **43.9 (39.1, 48.7)** | **20.2 (16.6, 23.9)** |
| **Age group** |  |  |  |
| 15-16 years | 37.6 (31.4, 43.8) | 46.2 (39.9, 52.5) | 16.2 (12.4, 19.9) |
| 17 years | 32.3 (25.5, 39.1) | 39.0 (32.7, 45.4) | 28.7 (22.0, 35.3) |
| **Sex** |  |  |  |
| Male | 37.3 (31.3, 43.2) | 43.0 (35.7, 50.4) | 19.7 (14.9, 24.4) |
| Female | 34.5 (28.1, 40.9) | 44.7 (38.0, 51.4) | 20.8 (16.0, 25.6) |
| **Remoteness** |  |  |  |
| Major cities | 35.7 (30.6, 40.9) | 42.1 (36.5, 47.6) | 22.2 (17.7, 26.6) |
| Inner regional | 36.7 (28.2, 45.2) | 48.6 (39.7, 57.6) | 14.6 (9.2, 20.0) |
| Outer regional, remote | 34.5 (21.1, 48.0) | 44.1 (30.2, 57.9) | 21.4 (9.4, 33.4) |
| **Education status** |  |  |  |
| Currently studying | 35.6 (31.1, 40.2) | 44.2 (39.3, 49.2) | 20.1 (16.4, 23.9) |
| Not currently studying | 38.0 (22.4, 53.7) | 40.8 (23.4, 58.2) | 21.2 (11.8, 30.6) |
| **2011-12 NHS^5^** | **30.0 (26.3, 33.8)** | **49.9 (46.0, 53.7)** | **20.1 (17.2, 23.0)** |
| **Age group** |  |  |  |
| 15-16 years | 34.6 (29.7, 39.6) | 50.9 (45.6, 56.3) | 14.4 (10.9, 17.9) |
| 17 years | 22.1 (15.9, 28.4) | 48.0 (40.7, 55.3) | 29.9 (22.6, 37.1) |
| **Sex** |  |  |  |
| Male | 31.7 (25.3, 38.2) | 46.6 (40.2, 53.1) | 21.6 (16.5, 26.8) |
| Female | 28.2 (23.2, 33.2) | 53.3 (48.1, 58.5) | 18.5 (14.9, 22.1) |
| **Remoteness** |  |  |  |
| Major cities | 28.8 (24.8, 32.8) | 50.4 (46.5, 54.3) | 20.8 (17.5, 24.1) |
| Inner regional | 30.5 (19.8, 41.3) | 53.8 (42.5, 65.2) | 15.6 (8.6, 22.7) |
| Outer regional, remote | 37.0 (26.4, 47.5) | 38.8 (26.7, 50.9) | 24.2 (14.0, 34.5) |
| **Education status** |  |  |  |
| Currently studying | 29.8 (25.9, 33.8) | 50.9 (46.6, 55.2) | 19.3 (16.4, 22.2) |
| Not currently studying | 31.8 (18.4, 45.1) | 40.4 (26.9, 53.9) | 27.9 (15.1, 40.7) |
| **2014-15 NHS^6^** | **38.6 (33.7, 43.4)** | **42.3 (38.4, 46.1)** | **19.2 (15.0, 23.3)** |
| **Age group** |  |  |  |
| 15-16 years | 38.5 (32.6, 44.5) | 46.5 (41.4, 51.5) | 15.0 (10.7, 19.3) |
| 17 years | 38.6 (29.6, 47.6) | 34.2 (27.6, 40.9) | 27.2 (19.4, 34.9) |
| **Sex** |  |  |  |
| Male | 42.2 (35.4, 49.0) | 40.7 (34.6, 46.7) | 17.1 (12.0, 22.3) |
| Female | 34.8 (27.2, 42.4) | 43.9 (37.7, 50.1) | 21.3 (15.1, 27.4) |
| **Remoteness** |  |  |  |
| Major cities | 37.7 (32.1, 43.3) | 44.6 (39.9, 49.4) | 17.7 (12.9, 22.4) |
| Inner regional | 37.3 (23.4, 51.3) | 39.1 (28.9, 49.3) | 23.6 (12.4, 34.8) |
| Outer regional, remote | 46.3 (31.9, 60.7) | 33.8 (23.0, 44.6) | 19.9 (6.8, 33.0) |
| **Education status** |  |  |  |
| Currently studying | 38.9 (34.4, 43.4) | 43.0 (39.1, 46.8) | 18.1 (14.0, 22.2) |
| Not currently studying | 33.0 (8.4, 57.7) | 31.7 (10.7, 52.7) | 35.3 (8.3, 62,3) |
| **2017-18 NHS^7^** | **47.8 (43.7, 51.9)** | **39.6 (36.0, 43.2)** | **12.6 (9.7, 15.6)** |
| **Age group** |  |  |  |
| 15-16 years | 48.8 (43.3, 54.4) | 40.7 (35.6, 45.8) | 10.4 (7.2, 13.6) |
| 17 years | 45.6 (38.0, 53.3) | 37.3 (29.7, 44.9) | 17.0 (11.1, 22.9) |
| **Sex** |  |  |  |
| Male | 50.5 (44.6, 56.5) | 36.2 (30.3, 42.2) | 13.3 (9.5, 17.0) |
| Female | 44.9 (39.9, 49.9) | 43.2 (37.6, 48.8) | 11.9 (7.8, 16.0) |
| **Remoteness** |  |  |  |
| Major cities | 45.0 (40.1, 49.9) | 41.9 (37.3, 46.4) | 13.2 (9.3, 17.1) |
| Inner regional | 57.8 (46.7, 68.9) | 31.9 (23.2, 40.6) | 10.3 (5.4, 15.3) |
| Outer regional, remote | 48.8 (35.5, 62.1) | 38.3 (24.8, 51.8) | 13.0 (5.2, 20.7) |
| **Education status** |  |  |  |
| Currently studying | 48.3 (44.1, 52.4) | 39.5 (35.9, 43.0) | 12.2 (9.2, 15.3) |
| Not currently studying | 41.4 (27.5, 55.3) | 41.1 (24.0, 58.1) | 17.6 (5.5, 29.6) |

-- indicates that data were not presented due to small numbers in one or more categories.

CI, confidence intervals; NATSIHS, National Aboriginal and Torres Strait Islander Health Survey; NHS, National Health Survey.

1. Australian Bureau of Statistics, Australian Aboriginal and Torres Strait Islander Health Survey (Core component) 2012-13, (accessed through Australian Bureau of Statistics DataLab July 2021).

2. Australian Bureau of Statistics, Australian Statistical Geography Standard Remoteness Structure, ABS. <https://www.abs.gov.au/websitedbs/d3310114.nsf/home/remoteness+structure> (accessed July 2021).

3. Australian Bureau of Statistics, National Aboriginal and Torres Strait Islander Health Survey, 2018-19, (accessed through Australian Bureau of Statistics DataLab July 2021).

4. Australian Bureau of Statistics, National Health Survey 2007-08, (accessed through Australian Bureau of Statistics DataLab July 2021). 5. Australian Bureau of Statistics, National Health Survey 2011-12, (accessed through Australian Bureau of Statistics DataLab July 2021).

6. Australian Bureau of Statistics, National Health Survey 2014-15, (accessed through Australian Bureau of Statistics DataLab July 2021).

7. Australian Bureau of Statistics, National Health Survey 2017-18, (accessed through Australian Bureau of Statistics DataLab July 2021).

**Table S3. National prevalence of youth (15-17 years) current, ex-, never and ever smokers, and Adjusted Prevalence Ratios (aPR) for ‘ever smoked’, overall and by respondent type, using weighted data**

|  | **Smoking status Weighted % (95% CI)** |  |  |  | **Ever smoked** |
| --- | --- | --- | --- | --- | --- |
|  | **Current smoker** | **Ex-smoker** | **Never smoker** | **Ever smoker** | **aPR (95% CI)** |
| **Aboriginal and Torres Strait Islander population** |  |  |  |  |  |
| **2012-13 AATSIHS^1^** |  |  |  |  |  |
| Total | 18.9 (15.4, 22.3) | 4.3 (2.1, 6.4) | 76.8 (72.9, 80.8) | 23.2 (19.2, 27.1) | - |
| Proxy | 16.4 (11.0, 21.8) | **--** | -- | -- | 1 (Ref) |
| Youth self-report with parent present | 17.5 (11.6, 23.4) | 5.6 (12.2, 10.1) | 76.8 (70.0, 83.6) | 23.2 (16.4, 30.0) | 1.19 (0.79, 1.80) |
| Youth self-report independently | 25.2 (16.3, 34.1) | **--** | -- | -- | 1.50 (0.95, 2.36) |
| **2018-19 NATSIHS^2^** |  |  |  |  |  |
| Total | 12.7 (8.4, 17.0) | 3.7 (0.8, 6.6) | 83.6 (78.5, 88.8) | 16.4 (11.2, 21.5) | - |
| Proxy | 11.6 (6.1, 17.1) | **--** | -- | -- | 1 (Ref) |
| Youth self-report with parent present | 10.2 (3.8, 16.6) | 3.7 (0.0, 7.8) | 86.0 (78.3, 93.7) | 14.0 (78.4, 93.7) | 0.94 (0.40, 2.19) |
| Youth self-report independently | 34.4 (6.0, 62.8) | **--** | -- | -- | 3.43 (1.51, 7.79) |
| **Total population** |  |  |  |  |  |
| **2007-08 NHS^3^** |  |  |  |  |  |
| Total | 6.9 (4.6, 9.1) | 3.6 (1.9, 5.2) | 89.6 (86.9, 92.3) | 10.4 (7.7, 13.1) | - |
| Proxy | 3.6 (1.1, 6.1) | -- | -- | -- | 1 (Ref) |
| Youth self-report with parent present | 4.8 (1.8, 7.8) | -- | -- | -- | 1.41 (0.63, 3.17) |
| Youth self-report independently | 17.2 (8.7, 25.6) | 3.7 (1.3, 6.1) | 79.1 (70.9, 87.4) | 20.9 (12.6, 29.1) | 3.03 (1.32, 6.98) |
| **2011-12 NHS^4^** |  |  |  |  |  |
| Total | 6.7 (4.7, 8.6) | 4.1 (2.3, 5.9) | 89.2 (87.1, 91.4) | 10.8 (8.6, 12.9) | - |
| Proxy | 5.4 (2.0, 8.4) | -- | -- | -- | 1 (Ref) |
| Youth self-report with parent present | 4.8 (2.5, 7.1) | -- | -- | -- | 1.68 (0.80, 3.53) |
| Youth self-report independently | 13.2 (7.2, 19.2) | 5.2 (2.0, 8.4) | 81.6 (75.3, 87.9) | 18.4 (12.1, 24.7) | 2.89 (1.34, 6.25) |
| **2014-15 NHS^5^** |  |  |  |  |  |
| Total | 3.5 (2.0, 4.9) | 1.7 (0.7, 2.7) | 94.9 (93.1, 96.7) | 5.1 (3.3, 6.9) | - |
| Proxy | -- | -- | 96.0 (93.4, 98.5) | 4.0 (1.5, 6.6) | 1 (Ref) |
| Youth self-report with parent present | -- | -- | 95.8 (93.6, 97.9) | 4.2 (2.1, 6.4) | 1.09 (0.48, 2.47) |
| Youth self-report independently | -- | -- | 90.7 (84.9, 96.5) | 9.3 (3.5, 15.1) | 1.75 (0.78, 3.93) |
| **2017-18 NHS^6^** |  |  |  |  |  |
| Total | 3.2 (1.4, 5.0) | 2.3 (1.1, 3.4) | 94.5 (92.3, 96.7) | 5.5 (3.3, 7.7) | - |
| Proxy | -- | -- | 95.4 (93.1, 97.8) | 4.6 (2.2, 6.9) | 1 (Ref) |
| Youth self-report with parent present | -- | -- | 95.5 (92.1, 98.8) | 4.5 (1.2, 7.9) | 1.00 (0.36, 2.79) |
| Youth self-report independently | -- | -- | 88.2 (79.6, 96.7) | 11.8 (3.3, 20.4) | 2.24 (1.09, 4.59) |

aPR = Prevalence Ratio adjusted for age, sex, remoteness, and education status. Prevalence estimates and Prevalence Ratios are calculated using weighted data.

-- indicates that data were not presented due to small numbers in one or more categories.

CI, confidence intervals; NATSIHS, National Aboriginal and Torres Strait Islander Health Survey; NHS, National Health Survey.

1. Australian Bureau of Statistics, Australian Aboriginal and Torres Strait Islander Health Survey (Core component) 2012-13, (accessed through Australian Bureau of Statistics DataLab July 2021).

2. Australian Bureau of Statistics, National Aboriginal and Torres Strait Islander Health Survey, 2018-19, (accessed through Australian Bureau of Statistics DataLab July 2021).

3. Australian Bureau of Statistics, National Health Survey 2007-08, (accessed through Australian Bureau of Statistics DataLab July 2021).

4. Australian Bureau of Statistics, National Health Survey 2011-12, (accessed through Australian Bureau of Statistics DataLab July 2021).

5. Australian Bureau of Statistics, National Health Survey 2014-15, (accessed through Australian Bureau of Statistics DataLab July 2021).

6. Australian Bureau of Statistics, National Health Survey 2017-18, (accessed through Australian Bureau of Statistics DataLab July 2021).

**Table S4. Current or previous Australian surveys collecting data on youth smoking**

| **Survey** | **Smoking questions asked in youth (15-17yrs)?** | **Sample** | **Representative of sample population** | **Respondent options** | **Scope limitations** |
| --- | --- | --- | --- | --- | --- |
| **National Aboriginal and Torres Strait Islander Health Surveys (NATSIHS)** | Yes, from 2012-13 | Aboriginal and Torres Strait Islander participants, all ages | Yes | Parent (or proxy) answered questions; parent present for all smoking questions; parent present for some smoking questions; parent not present for smoking questions |  |
| **National Health Surveys (NHS)** | Yes, from 2007-08 | Australian participants, all ages | Yes | Parent (or proxy) answered questions, Parent present for all smoking questions, Parent present for some smoking questions, Parent not present for smoking questions |  |
| **National Aboriginal and Torres Strait Islander Social Survey (NATSISS)** | Yes, from 2002 | Aboriginal and Torres Strait Islander participants, all ages | Yes | Not found in publicly available data item lists. |  |
| **National Aboriginal and Torres Strait Islander Survey, 1994** | Yes | Aboriginal and Torres Strait Islander participants, all ages | Yes | Not found in publicly available data item lists. |  |
| **General Social Survey 2019** | Yes | Australian participants, all ages | Yes | Not found in publicly available data item lists. |  |
| **Young Minds Matter: the second Australian Child and Adolescent Survey of Mental Health and Wellbeing 2015** | Yes | Australian participants aged 4-17 years | Yes | All persons 11+ years self-reported with parent/carer consent. Consenting youth were given the use of a CASI tablet computer and, wherever possible, completed the questionnaire in private at the same time that the parent or carer was being interviewed in person. | Cannot be used for Aboriginal and Torres Strait Islander specific estimates due to small sample. |
| **National Survey of Mental Health and Wellbeing** | Partly from 2007, 16+ | Australian participants aged 16-85 years | Yes | Proxies were not used. All interviews were conducted in private where possible. Some may have been conducted in presence of other household members, according to the wishes of the respondent. |  |
| **National Drug Strategy Household Survey** | Yes | Australian participants aged 14+ years (12+ prior to 2019) | Yes | No proxy, but surveys could be conducted in the presence of others. From 2007 to 2016, parent presence at the surveys with youth aged 12-13 years ranged from 58% to 63% (1,2). In the 2019 NDNHS, parent presence for youth aged 14-15 years was around 40% (3). | Cannot be used for Aboriginal and Torres Strait Islander specific estimates due to small sample. |
| **Australian secondary school students alcohol and drug (ASSAD) survey** | Yes | Australian participants aged 12-17 years attending school | Yes | All participants self-report at school, using pen and paper survey. Teachers could be present in room but did not assist with survey administration. | Does not adequately capture youth living in remote settings, or attending small schools (fewer than 100 students). |
| **The Mayi Kuwayu: the National Study of Aboriginal and Torres Strait Islander wellbeing** | Partly, 16+ | Aboriginal and/or Torres Strait Islander participants aged 16 years and over | No | Most participants completed the mail survey using paper and pen. Participants also had the option to complete the survey online or over the phone, with face-to-face surveying occurring in selected areas. No information on whether anyone else was present at the time was taken in the survey. | Not currently designed to generate nationally representative estimates. |

1. Australian Institute of Health and Welfare. National Drug Strategy Household Survey 2016. Australian Institute of Health and Welfare. Available through ADA Dataverse <https://dataverse.ada.edu.au/dataset.xhtml?persistentId=doi:10.4225/87/JUDY2Y> (accessed July 2021).

2. Australian Institute of Health and Welfare. National Drug Strategy Household Survey 2007. Australian Institute of Health and Welfare. Available through ADA Dataverse https://dataverse.ada.edu.au/dataset.xhtml?persistentId=doi:10.4225/87/T7FITH (accessed July 2021).

1. Australian Institute of Health and Welfare. National Drug Strategy Household Survey 2019. Australian Institute of Health and Welfare. Available through ADA Dataverse https://dataverse.ada.edu.au/dataset.xhtml?persistentId=doi:10.26193/WRHDUL (accessed July 2021).
